# Development and preliminary psychometric investigation of the German Satisfaction with Comprehensive Cancer Care (SCCC) Questionnaire

**DOI:** 10.1186/s12955-021-01784-y

**Published:** 2021-05-17

**Authors:** Peter Esser, Leon Sautier, Susanne Sarkar, Georgia Schilling, Carsten Bokemeyer, Uwe Koch, Michael Friedrich, Gautier Defossez, Anja Mehnert-Theuerkauf

**Affiliations:** 1 Department of Medical Psychology and Medical Sociology, University Medical Center Leipzig, Leipzig, Germany; 2 Department of Medical Psychology, University Medical Center Hamburg-Eppendorf, Hamburg, Germany; 3 Clinical Psychology and Psychotherapy, Institute of Psychology, Faculty of Psychology and Movement Sciences, University of Hamburg, Hamburg, Germany; 4 “Hubertus Wald” Tumor Centre, University Cancer Center Hamburg (UCCH), University Medical Center Hamburg-Eppendorf, Hamburg, Germany; 5 Department of Clinical Oncology, Asklepios Tumorzentrum Hamburg, Hamburg, Germany; 6 Department of Internal Medicine II, University Medical Center Hamburg-Eppendorf, Hamburg, Germany; 7 UFR Médecine Et Pharmacie, Université de Poitiers, Poitiers, France

**Keywords:** Patient satisfaction, Cancer, Neoplasm, Validation, Patient-reported outcomes

## Abstract

**Purpose:**

The assessment of patient satisfaction during treatment is essential to provide patient-centered high-quality cancer care. Nevertheless, no German instrument assesses patient satisfaction with comprehensive cancer care, which not only includes oncological treatment, but also interpersonal quality of care as well as psychosocial support services. Based on the French REPERES-60, we developed the German Patient Satisfaction with Comprehensive Cancer Care (SCCC) questionnaire.

**Methods:**

The REPERES-60 was translated and the items were adapted to make it applicable to the German healthcare system and across different tumor entities. Scales of the resulting instrument were extracted via principal axis factoring (PAF). Subsequently, we investigated the reliability (Cronbach’s Alpha, CA), discriminatory power (corrected item-scale correlations) and convergent validity (pre-specified correlations of the SCCC with different outcomes).

**Results:**

The SCCC consisted of 32 items which were subsequently tested among a sample of 333 patients across different tumor entities (response rate: 47%). Average age was 59 years (standard deviation: 14), 63% were male. PAF revealed four multi-item scales named *Competence*, *Information*, *Access* and *Support* accounting for 71% of the variance. Two single-items scales assess *global satisfaction with medical and psychosocial care*, respectively. CA across the multi-item scales ranged from .84 to .96. Discriminatory power was sufficiently high, with all *r* ≥ .5. Convergent validity was largely verified by negative associations of the four multi-item scales with depressive/anxious symptomatology (*r* ≥ − .18, *p* < .01) and fatigue/overall symptom burden (*r* ≥ − .14, *p* < .01).

**Conclusion:**

We developed a tool to assess patient satisfaction with comprehensive cancer care in Germany. The SCCC showed satisfactory psychometric properties. Further studies are needed to verify these preliminary findings.

**Supplementary Information:**

The online version contains supplementary material available at 10.1186/s12955-021-01784-y.

## Plain English Summary

### What is the problem?

The assessment of patient satisfaction during their oncological treatment is important for oncological staff and health care institutions to optimize the care they provide. To date, German instruments on patient satisfaction rather focus on the medical treatment of the disease. However, psychosocial aspects during the treatment process such as human quality of the medical staff, interpersonal aspects between the patients and the medical staff or access to psychosocial support services are often not adequately measured.

### What did we do?

We used an existing questionnaire in French language (REPERES-60) as a starting point. Subsequently, we adapted, removed and added items to ensure applicability to the German health care system. The final questionnaire was filled in by more than 300 cancer patients. Based on their responses, statistical tests were applied to check whether quality criteria were met.

### What did we find?

The *Satisfaction with Comprehensive Cancer Care* (SCCC) questionnaire contains 32 items. The items can be structured in four multi-item scales (*Competence*, *Information*, *Access* and *Support)* and two single-items scales (G*lobal *satisfaction* with medical care* and *Global satisfaction with psychosocial care)*. The statistical tests showed that the quality criteria were satisfactorily met.

### What do we conclude?

The questionnaire showed satisfactory psychometric properties. However, future studies are needed to validate our preliminary findings.

## Introduction

The patient perspective during oncological treatment is considered essential to provide patient-centered high quality cancer care [1]. In fact, studies demonstrate that such subjective evaluations of care are associated with better quality of life and treatment compliance among patients [2, 3]. Accordingly, the assessment of patient satisfaction receives increasing attention within the field of clinical oncology [3].

Existing instruments on patient satisfaction with cancer care considerably vary in their scope and length [4–9]. One of the most widely used instruments is the 32-item *In-Patient Satisfaction with Care* (IN-PATSAT-32) questionnaire [8], which assesses satisfaction of inpatients with respect to doctors, nurses and the organization within the hospital. The detailed 120-item *Patient Satisfaction and Quality of life in Oncological Care* (PASQOC) questionnaire [6] in turn focuses on outpatient cancer care and assesses multiple aspects such as treatment environment. The 16-item *Family Satisfaction with Advanced Cancer Care-Patient Version* (FAMCARE-P) questionnaire [9] was developed for use in palliative care and addresses specific issues within the palliative setting such as inclusion of the family members into medical decisions or information about the prognosis.

Even though most of these aforementioned instruments measure interpersonal competence of the clinical staff, they mainly focus on the satisfaction with medical treatment and do not explicitly assess the satisfaction with psychosocial support services. Since professional psycho-social support should be part of comprehensive cancer care [10, 11], the evaluation of such supportive services beyond the actual medical treatment seems warranted.

A patient satisfaction questionnaire which also assesses the quality of and access to psycho-social support is the French REPERES-60 (*Recherche Evaluative sur la Performance des Réseaux de Santé*) questionnaire [12]. The domains of this questionnaire were developed based on extensive literature review and findings from focus groups with patients and experts [12]. Items were selected according to quantitative analyses conducted among a sample of 820 breast cancer patients [12]. Nevertheless, the REPERES-60 was developed within the French health care system and was tailored to the specific needs of breast cancer patients. This in turn may limit the applicability of this questionnaire for its use in other health care systems and among other patient groups.

To date, no questionnaire comparable to the REPERES-60 is available in German language. Such an assessment tool may help German speaking cancer patients to evaluate whether and to what extent their expectations and needs during treatment were met. Health care policy in turn may use such ratings to adapt their comprehensive care programs accordingly. For this purpose, we translated the REPERES-60 and used it as a basis to develop the German *Satisfaction with Comprehensive Cancer Care* (SCCC) questionnaire.

## Methods

### Study design

This cross-sectional data was collected between December 2012 and December 2013 within a pilot study for a intervention study evaluating an electronic psycho-oncological adaptive screening program. Individuals were eligible if they were (i) diagnosed with any malignancy according to ICD-10, (ii) 18 years or older and (iii) able to read and speak German. Patients were excluded if they had any physical and/or cognitive impairments potentially impeding the ability to give informed consent.

Eligible patients who were identified via review of their medical records and who approved to be contacted by medical staff were consecutively approached by research assistants at the in- and outpatient facilities of the Hubertus Wald Tumorzentrum of the University Cancer Center of Hamburg-Eppendorf (UCCH). Patients agreeing to participate were given the set of questionnaires together with a pre-stamped return envelope. All patients provided written informed consent prior to study participation. The study was approved by the ethics committee of the local General Medical Council of Hamburg (PV4371).

Additional details can be found in a previous project publication [13].

### Basis for the SCCC (REPERES-60)

The REPERES-60 [12] served as a starting point to develop the SCCC. The 60 items across 13 subscales assess *access to primary care* (ASP), *access to secondary care* (ASS), *competence and communication skills of primary care doctors* (COMG), *competence of secondary care doctors* (COMPS), *communication skills of secondary care doctors* (COMMS), *choice among doctors* (PC), *human qualities of doctors* (QH), *global satisfaction* (SG), *cover for medical expenses* (CM), *listening abilities and information provided by doctors* (INF), *organization and follow-up of medical care provision* (ORG), *psychological support* (PSY) and *material environment* (ENV). Items are rated on a five-point Likert scale ranging from 1 (*bad/do not agree at all*) to 5 (*excellent/completely agree*). The scales INF and ORG contain an additional response option (*not concerned).*

### Development of the SCCC

First, the REPERES-60 was translated into German by a bilingual native speaker. The translations were subsequently discussed within the working group and linguistically modified wherever considered necessary.

After translation of the REPERES-60, the items were adapted and developed with the aim to make the instrument (i) applicable to the German healthcare system, (ii) usable across different tumor entities and (iii) comprehensive by addressing all medical and psychosocial aspects warranted within comprehensive cancer care.

Twenty-eight items across the subscales ASP, ASS, PC, CM, ORG and ENV were either not of significant relevance for patients in the German healthcare system (e.g., degree to which medical expenses are paid by the insurance) or assessed rather formal criteria of medical treatment (e.g., the number of specialists that can be consulted/lengths of waiting time at different stages of treatment). To focus on the quality of the actual medical treatment and psychosocial aspects within the oncological care, these items were removed. We further excluded two items of the subscale INF which were breast-cancer specific (explanations prior to breast surgery/information on breast reconstruction). By merging the different types of physicians of the REPERES-60 (oncologists/surgeons vs. general practitioner/gynecologist) into the broader term “physicians”, another 6 items could be excluded from the item pool given that certain questions in COMG and COMMS only differed by the type of care provider (COMG: general practitioner/gynecologist; COMMS: oncologist/surgeon).

The questionnaire should also assess global patient satisfaction, but separately for medical and psychosocial care. Therefore, we excluded three of the four items of the global satisfaction scale of the REPERES-60 (*the care I receive is practically perfect*; *I am dissatisfied with some things in the care I receive; some things in the care I receive could be better*) and split the remaining item *I am very satisfied with the care I received* in 2 items, separately assessing global satisfaction with medical and psychosocial care.

To assess all aspects of comprehensive cancer care and to adapt the assessment to available services within the German health care system, we further added 10 items. In detail, the items addressed *human quality of physicians* (interest of physicians towards patient and in his/her psychosocial condition), *information* (psychosocial support services/alternative possibilities of treatment and their consequences) and *accessibility to psychosocial services* (psychosocial counselling on supportive care services/psycho-oncological support/legal and financial advice/pastoral support/support in searching for a psychotherapist/support in acquiring coping strategies/support to join self-help or therapeutic groups). These items were developed within the working group, which encompassed expertise in both research and clinical practice. The items to assess the accessibility of psychosocial services should represent the services that may be available in German comprehensive cancer care centers.

Finally, the adaptation process resulted in a total of 32 items. 30 items were rated on a 5-point scale ranging from 1 (bad) to 5 (excellent); the 2 global items were rated from 1 (do not agree at all) to 5 (completely agree). Higher values indicate higher satisfaction. The seven items related to accessibility to psychosocial care services (see Table 2, items 19–25) contained the additional response option 0 (not applicable).

### Validation instruments

*Depressive* and *anxious symptomatology* was assessed with the validated German version of the Patient Health Questionnaire (PHQ) [14]. In detail, we used the modules on depressive (9-item PHQ-9) and anxious (7-item GAD-7) symptomatology. The items within these modules correspond with the criteria for major depression (PHQ-9) and generalized anxiety disorder (GAD-7) as defined in the fourth edition of the *Diagnostic and Statistical Manual of Mental Disorders* (DSM-IV). Patients rate the frequency of respective symptoms within the last two weeks on a four-point Likert scale ranging from 0 (not at all) to 3 (nearly every day). Higher values indicate higher depressive and anxious symptomatology, respectively.

*Overall symptom burden and fatigue:* To assess overall symptom burden, we calculated the mean score across the 20 physical symptoms of the problem list within the Distress thermometer (DT), which is an internationally established and comprehensive tool to assess physical and psychosocial distress among cancer survivors [15]. In contrast to the original version, symptoms could be rated on a four-point Likert scale ranging from 0 (not at all) to 3 (strong), with higher values indicating higher symptom burden. Of this list, we also selected fatigue as single item.

### Sociodemographic and medical data

Sociodemographic data were collected by self report and medical data was transferred from the medical records.

### Statistical analysis

Descriptive statistics were used to provide medical and sociodemographic sample characteristics.

For the 32 items of the SCCC, we provided mean and standard deviation as well as skewness and kurtosis. Values of skewness > 2 and kurtosis > 7 were used to indicate substantial deviations from normal distribution [16]. To further assess applicability of the items, we investigated (i) the distribution of response choices for each item to identify any floor and ceiling effects (according to Defossez et al. [12] indicated if > 50% filled in the same response option) and (ii) the respective number of missing data.

Dimensions were extracted via principal axis factoring (PAF). Varimax rotation was used to ease interpretation of the results. Since multivariate outliers may bias factor and reliability analysis [17, 18], we identified such outliers among the 182 cases with complete questionnaire data via Mahalanobis distance exceeding the critical value of the chi-square distribution (p < 0.001). In this subsample, only one outlier was found and kept in the sample. Sampling adequacy was further confirmed via acceptable values of Kaiser–Meyer–Olkin (KMO = 9.5) and Bartlett’s test of sphericity (χ^2^ (435) = 6099, p < 0.001).

Factors were selected based on Kaiser’s Criterion retaining all factors with an eigenvalue above 1. The 2 global satisfaction items were excluded from PAF and treated as single item-scales since their total evaluation of medical/psychosocial care was assumed to be partly included within each specific area and thus an assignment of these items to any subscale did not seem reasonable. Assignment of items to the factors should be primarily based on the size of the loadings, but also needed to correspond with the content of the respective scale.

Reliability was assessed via Cronbach’s Alpha (CA). Additionally, we calculated the average inter-item correlation which should optimally lie in the interval between 0.2 and 0.4 [19]. Discriminatory power was investigated via corrected item-scale-correlations which should be equal or greater than 0.3 [20]. We also present the correlations between the scales to assess the strength of respective relationships.

To assess convergent validity, we tested pre-specified associations of the SCCC scales with external criteria. Previous research indicates that patient satisfaction is positively associated with quality of life (QoL) [2, 3, 21]. Therefore, we selected four outcomes that assess physical and psychological components of QoL for which associations with patient satisfaction have been demonstrated in previous analyses, i.e., anxiety, depression, symptom burden and fatigue [9, 21]. Given that higher values in our validation criteria indicate higher symptomatology (and thus worse QoL), we expected that all of these variables are negatively correlated with the SCCC scales. Furthermore, we also selected the sociodemographic variable age, for which previous studies showed positive associations with patient satisfaction [22, 23].

During analyses, we found an increased number of missing values in the 7 variables containing the response option “not applicable”. Therefore, it could not be definitely decided whether these missing values were really missing or neglected precisely because they were inapplicable to the patients. To avoid any misinterpretation of data, we decided for a conservative approach and merged the two categories “missing” and “not applicable” into “missing” for all subsequent analyses.

Patients with more than 50% missing data in the SCCC were excluded. Means of outcomes were calculated if at least 50% of the items had valid values. Effect sizes were interpreted according to Cohen (small: r ≥ 0.1; medium: r ≥ 0.3; large: r ≥ 0.5) [24]. Given the high amount of missing data in the 7 items containing the response option “not applicable”, we applied pairwise correlations for both PAF and correlation analyses. We used two tailed tests with an alpha of 0.05. Analyses were conducted with IBM SPSS Statistics (Version 26) and R (version 3.5.0, the R Foundation for Statistical Computing).

## Results

Of 885 screened patients, 174 patients did not meet inclusion criteria. Of the 711 eligible patients, 583 agreed to participate. Of those willing to participate, 248 patients did not (completely) return the set of questionnaires and two patients were further excluded due to too many missing values in the SCCC, resulting in a final number of 333 patients (response rate: 47%). The mean age was 59 years (standard deviation: 14), about two thirds were male, hematological malignancies were most frequent (36%) (Table 1).

**Table 1:** Sociodemographic and medical sample characteristics (N = 333)

	n (valid %)
*Age in years (M, SD)*	59 (14)
*Age group*	
18 to < 40	33 (10)
40 to < 50	42 (13)
50 to < 60	81 (24)
60 to < 70	95 (29)
70 to < 90	82 (25)
*Gender*	
Female	122 (37)
Male	211 (63)
*In partnership*	214 (78)
Yes	
*Education*	
≤ 10 years	199 (61)
> 10 years	122 (37)
Others^a^	8 (2)
*Diagnostic groups*	
Breast/gynecological (C50-53, C58)	12 (4)
Prostate/male genital organs (C61-62)	15 (5)
Hematologic (C81-85, C90-92)	121 (36)
Urinary tract (C64-68, C74)	18 (5)
Gastrointestinal (C15-16, C18-20)	35 (11)
Pancreas (C25)	20 (6)
Lung (C34, C49)	22 (7)
Others	73 (22)
*Relapse*	
Yes	71 (23)
*Months since current diagnosis* (M, SD)	11 (18)
*Treatment*^*b*^	
Surgery	142 (46)
Radiotherapy	88 (28)
Chemotherapy	252 (81)
Stem cell transplantation	14 (5)
*Karnofsky index* (M, SD)	96 (8)

Results slightly differ from a previous publication among this sample [13] due to exclusion of 2 patients, different merging of categories and reconstruction of missing data

^a^No educational degree/currently student

^b^Ongoing/completed

Both skewness (between − 1.5 and 0.04) and kurtosis (between 2.0 and 6.0) did not indicate substantial deviations from normal distribution (Table 2). The *Global Medical Satisfaction* item showed the most extreme values in both skewness and kurtosis, with the majority of responses in the high range of the scale. Nevertheless, no floor or ceiling effects could be identified. Missing values were low for the majority of items (0–6%) except for the 7 items containing the response option “not applicable” (8–23%).

**Table 2:** Item characteristics and factor loadings of the SCCC among 333 cancer survivors

*Satisfaction with…*		Item characteristics				DP	Factor analyses			
		M (SD)	SKE	KUR	MIS N (%)		F1	F2	F3	F4
*Competence and human quality of physician (competence)*^*a*^										
1	Care and thoroughness in examinations/accuracy of diagnoses	3.7 (0.9)	− 0.6	3.1	3 (1)	.82	**.81**	.24	.16	.12
2	Skills and experience	3.9 (0.9)	− 0.4	2.9	4 (1)	.79	**.80**	.22	.14	.08
3	Care and thoroughness in treatment throughout the illness	3.8 (1.0)	− 0.7	3.1	3 (1)	.84	**.79**	.25	.13	.26
4	Explanations on medical procedures and tests	3.5 (0.9)	− 0.3	2.8	0 (0)	.78	**.67**	.42	.18	.17
5	Attention to patient opinion	3.4 (1.0)	− 0.2	2.5	0 (0)	.84	**.71**	.36	.17	.27
6	Advice on preventative measures to stay healthy	3.0 (1.0)	0.0	2.7	8 (2)	.67	**.48**	.46	.18	.28
7	Kindness and courtesy	3.9 (0.9)	− 0.5	3.1	2 (1)	.81	**.77**	.22	.17	.22
8	Interest in person and health problems of the patient	3.7 (1.0)	− 0.5	2.9	2 (1)	.88	**.80**	.30	.16	.19
9	Interest in person and psychological condition	3.2 (1.0)	− 0.1	2.3	3 (1)	.77	**.59**	.32	.23	.36
10	Respect and attention to privacy	3.7 (0.9)	− 0.3	2.8	0 (0)	.83	**.71**	.31	.19	.27
11	Ability to reassure and support patients	3.5 (1.0)	− 0.3	2.7	1 (< 1)	.81	**.65**	.36	.15	.33
*Quality and quantity of information (information)*^*a*^										
12	Information on the disease	3.3 (0.9)	− 0.2	2.7	2 (1)	.81	.51	**.64**	.24	.12
13	Information on illness sequelae	3.0 (1.0)	< − 0.1	2.5	3 (1)	.84	.32	**.73**	.24	.20
14	Information on treatment as a whole	3.2 (0.9)	− 0.1	2.7	3 (1)	.83	.45	**.71**	.19	.15
15	Information on alternative treatment and its consequences	3.25 (1.0)	− 0.1	2.4	2 (1)	.81	.40	**.71**	.14	.20
16	Information on side effects of treatment	3.1 (1.0)	− 0.2	2.5	3 (1)	.81	.40	**.68**	.17	.19
17	Information on pain management	3.1 (1.0)	0.1	2.6	5 (2)	.77	.38	**.64**	.14	.25
18	Information on existing psychosocial support services	2.5 (1.0)	0.4	2.5	10 (3)	.65	.19	**.51**	.39	.36
*If needed: access to psychosocial support services (access)*^*a*^										
19	Access to psychosocial counselling on supportive care	2.9 (1.0)	0.1	2.9	28 (8)	.82	.24	.13	**.82**	.04
20	Access to psychological support	2.8 (1.0)	0.1	2.9	35 (11)	.78	.17	.11	**.83**	.08
21	Access to advice on legal and financial issues	2.7 (1.0)	0.2	2.7	43 (13)	.80	.07	.20	**.81**	.17
22	Access to pastoral support or company	2.9 (1.0)	0.2	2.8	64 (19)	.73	.11	.03	**.74**	.18
23	Support in searching for a psychotherapist	2.5 (1.0)	0.4	2.8	75 (23)	.76	.26	.35	**.60**	.31
24	Support in learning self-help strategies to cope with distress	2.3 (1.0)	0.4	2.7	77 (23)	.73	.16	.27	**.57**	.43
25	Access to self-help/therapeutic groups on specific topics	2.6 1.00)	0.3	2.8	73 (22)	.77	.11	.16	**.67**	.27
*Psychological support by medical staff (support)*^*a*^										
26	Opportunity to talk about health problems in the hospital	2.8 (1.0)	< − 0.1	2.3	19 (6)	.66	.24	.25	.33	**.52**
27	Opportunity to talk about health problems at home	3.4 (1.2)	− 0.3	2.3	12 (4)	.52	.23	.17	.25	**.35**
28	Psychological support provided by physicians	2.8 (1.0)	0.2	2.6	17 (5)	.69	.29	.34	.30	**.61**
29	Psychological support provided by nurses	3.2 (1.0)	< 0.1	2.4	14 (4)	.71	.28	.14	.24	**.72**
30	Assistance provided by medical staff throughout the care	3.5 (0.9)	− 0.2	2.7	10 (3)	.69	.47	.20	.19	**.55**
*Global medical satisfaction*^*b*^										
31	I am very satisfied with the medical care I receive	4.4 (0.8)	− 1.5	6.0	0 (0)	–	–	–	–	–
*Global psychosocial satisfaction*^*b*^						–	–	–	–	–
32	I am very satisfied with the psychosocial care I receive	3.1 (1.3)	− 0.3	2.0	18 (5)	–	–	–	–	–

Loadings in bold font indicate assignment to the factors;

M: mean, SD: standard deviation, DP: discriminatory power, i.e., corrected item-scale correlation; F1 to F4: extracted factors, values indicate factor loadings

^a^Scale ranged from 1 (bad) to 5 (excellent)

^b^Scale ranged from 1 (do not agree at all) to 5 (completely agree)

The rotated factor matrix resulted in 4 factors (Table 2) explaining 71% of the total variance. The assignment to the factors based on the maximum factor loading corresponded with the respective content of the scales. Therefore, no re-assignment seemed necessary. However, some items (e.g., items 6, 12, 24 and 30) loaded relatively high on more than one factor. The final subscales were named *Competence* (11 items; 51% of total variance), *Information* (7 items; 11% of total variance), *Access* (7 items; 5% of total variance) and *Support* (5 items; 4% of total variance); the two additional single-item scales were named *Global Medical Satisfaction* and *Global Psychosocial Satisfaction*.

CA and average inter-item correlations were high, i.e., 0.96 and 0.68 (*Competence)*, 0.93 and 0.66 (*Information)*, 0.93 and 0.64 (*Access)* and 0.84 and 0.53 (*Support)*, respectively. With all *r* ≥ 0.5, discriminatory power was sufficiently high across all items.

Correlations between the four multi-item scales were significant and large (*r* = 0.51 to 0.78). Correlations between the multi-item scales with the two single-item-scales considerably differed, ranging from medium to large effects (*r* between 0.31 and 0.62; Table 3). The smallest inter-scale correlation was found between the two single-item scales (*r* = 0.17).

**Table 3:** Correlations between the scales of the SCCC

	*Satisfaction with…*				
	Access	Support	Competence	Information	Global medical
Support	.59**				
Competence	.51**	.66**			
Information	.56**	.65**	.78**		
Global medical	.31**	.44**	.62**	.49**	
Global psychosocial	.41**	.37**	.32**	.36**	.17**

Effects can be interpreted as follows: 0.1 = small; 0.3 = medium, 0.5 = large

**p < .01

All but one SCCC scales showed small, but significant negative associations with levels of depressive and anxious symptomatology, overall symptom burden and fatigue (total values of *r* between 0.19 and 0.27; Table 4). No significant correlations were found between the SCCC scales and age. The scale *Global Psychosocial Satisfaction* did not show any correlations with the external criteria.

**Table 4:** Correlations of the scores of the SCCC with other outcomes to test convergent validity

	*Satisfaction with…*					
	Access	Support	Competence	Information	Global medical	Global psychosocial
Depressive symptomatology	− .21**	− .24**	− .20**	− .24**	− .18**	− .06
Anxious symptomatology	− .27**	− .20**	− .24**	− .24**	− .23**	− .10
Fatigue	− .18**	− .15**	− .14**	− .18**	− .16**	.07
Overall symptom burden	− .22**	− .17**	− .24**	− .20**	− .19**	− .03
Age	< − .01	− .09	− .03	.02	.05	< − .01

Effects can be interpreted as follows: 0.1 = small; 0.3 = medium, 0.5 = large

**p < .01

The final questionnaire is attached as Additional file 1: Table S1.

## Discussion

### Main findings

Based on the French REPERES-60, we developed a German questionnaire assessing patient satisfaction with comprehensive cancer care (SCCC). The SCCC revealed 4 multi-item dimensions named *Competence*, *Information*, *Access* and *Support* and two single-item scales named *Global Medical/Psychosocial Satisfaction*. Reliability and convergent validity were largely verified.

### Interpretation of the findings

Four multi-scale dimensions emerged measuring interpersonal and medical competence of the physicians (*Competence*), adequate magnitude and quality of disease-related information (*Information*), the possibility to use psychosocial services if needed (*Access*) and the level of psychological support (*Support*). It is to note that some items such as *advice on preventive measures* or *assistance provided by medical staff* loaded relatively similar on several factors, which in turn complicated the assignment to the factors from a statistical point of view. Nevertheless, all assignments based on the size of factor loadings corresponded with the content of the respective scale. Therefore, we assumed that the proposed structure of the items was justified based on the current data.

The two single-item scales *Global Medical/Psychosocial Satisfaction* were weakly correlated with each other. This implies that medical und psychosocial care may be differently evaluated and thus supports our methodological approach to use these two items as single-item scales. Furthermore, correlations between the multi-item scales and the two global items considerably differed. Based on the size of the correlations, it may be hypothesized that the aspect “Access” is most relevant for global satisfaction with psychosocial care, whereas competence of the physician may be of highest relevance for global satisfaction with medical care. Given the cross-sectional design, however, no causal conclusions can be drawn at this stage.

The average inter-item correlations were above the upper limit, which may indicate that some items may be redundant or the scales being too specific [19]. Furthermore, some items loaded relatively similar on several factors. One possibility may have been to delete respective items. After careful discussion, however, we decided against such a procedure at this stage: Our primary aim was to develop a questionnaire exhaustively assessing all relevant aspects of comprehensive cancer care in Germany. Items with high cross-loadings such as *advice on preventive measures* or *support in learning self-help coping strategies* were mostly neglected in previous assessment instruments, but are important aspects of care. Furthermore, the assignment to the factors for each item was verified “two-fold” (both with high factor loading and conceptual fit). Future studies among independent data sets should build upon these preliminary findings and test alternative models including shorter versions.

The scale *Global Medical Satisfaction* was highly negatively skewed, with many patients reporting very high ratings. It could be hypothesized that the response scale or the formulation of this item should be modified. Nevertheless, it is to note that we already assessed this issue with the superlative statement “being very satisfied with medical care”, which was intended to avoid an extreme positive rating. Therefore, this response pattern may be explained by social desirability, but may also reflect the high expertise provided by the medical staff in this specialized cancer center. In this context, we also note that no floor or ceiling effects across items were found, and that no item showed substantial deviations from normal distribution.

The number of missing values for items within the *Access* scale was considerably higher than across the rest of the items. One possible explanation is that these items were not applicable for a subset of patients, but that the majority of these patients did not use the “not applicable” option provided for these items and simply skipped them without any response. Originally, the extra response option “not applicable” was intended to avoid missing data and to be able to differentiate between “missing data” and “not applicable”. However, the results imply that this response option was not used by many participants and might be removed.

The fact that most of the SCCC scales were negatively correlated with physical and emotional symptom burden are in line with previous research showing that patient satisfaction is positively associated with higher quality of life [9, 21]. Therefore, these results largely verify convergent validity of the instrument. As an exception and in contrast to previous studies [22, 23], we did not find any association with age. This discrepancy, however, may be caused by different sample sizes. For example, the correlation in a previous study was similarly weak (*r* =  − 0.11), but reached significance due to large sample sizes (n = 7212) [4]. According to this assumption, a study using similar sample sizes than in our study (two subsamples with n = 453 and 438, respectively) did not find any significant associations with age either [5].

*Global Psychosocial Satisfaction* did not correlate with any of the validation outcomes. Previous studies which assessed global satisfaction did not separate between psychosocial and medical aspects of care (e.g., [3, 12]) and thus a comparison is limited. As a possible explanation for the result in our study, it may be assumed that patients did not know how to interpret the term “psychosocial care”. Therefore, concrete examples to illustrate this term may improve the validity of this item. The strongest inter-scale correlation was observed between *Competence* and *Information*. This may reflect the fact that some items may fit to both scales: For example, the *Competence*-item “explanations on medical procedures and tests” also address the aspect of information. Likewise, the *Information*-item “information on treatment as a whole” may also be considered relevant to evaluate the competence of a physician. Despite such ambiguities, a separate investigation of these two aspects seems warranted to ensure a differentiated evaluation of care.

Based on the considerations above, the final questionnaire (see Additional file 1: Table S1) does not contain any “not applicable”-option and includes illustrative examples for correct evaluation of the *Global Psychosocial Satisfaction* scale. Additionally, we decided to change the previous scale that ranged from “*bad”* to “*excellent”* into “*very bad”* to “*very good”*: This enabled symmetric response options on both the negative and positive spectrum and thus a clear neutral response option in the middle of the scale (named “average”). Finally, slight linguistic modifications were applied to enable uniform question formats within each scale.

### Clinical implications

Given that comprehensive cancer care is increasingly required in oncological health care facilities, patient evaluations from this tool may help health care policy to identify gaps in their care program which are overseen by previous questionnaires, e.g., regarding availability of psychosocial support services. In clinical practice, the monitoring of patient satisfaction with this comprehensive tool may help the medical team to identify starting points to improve the care they provide to their patients. In this context, we acknowledge that these preliminary validation results need to be confirmed in future studies.

### Strengths and limitations

To the best of our knowledge, we developed the first instrument in German language assessing patient satisfaction with all relevant aspects of comprehensive cancer care. The selection of items were either selected from the extensively developed and well-validated REPERES-60 [12] or carefully developed based on clinical considerations. Various validation criteria were selected according to previous literature to test the validity of the instrument.

As major limitation, we note that this study had an exploratory approach and thus may only provide preliminary findings: We primarily aimed to provide a set of items that exhaustively assesses all aspects which are relevant for comprehensive cancer care in Germany. Given this aim and the exploratory study design, we decided against elimination of items at this stage. Future confirmatory studies are needed to test the stability of this proposed structure and to investigate the potential superiority of alternative models including shorter versions. Such analyses, however, need independent samples and thus were beyond the scope of this preliminary validation. Furthermore, a longitudinal study will be needed to test its sensitivity to change. Even though the initial response rate was relatively high, only 47% of eligible patients provided enough data to be included in the analyses. Since non-responders were not systematically assessed, potential sample bias could not be identified. Even though such a sample bias may be problematic in epidemiological studies, we do not assume that any bias might have considerably changed the factor and correlation analyses used in this study. We assumed that the high number of missing values across the items of the *Access*-scale were caused by the “not applicable”-option. Based on this hypothesis, we decided to treat each “non-applicable”-response as missing, which may have biased the findings. By deleting this response option in the final version of the SCCC, however, further inconclusiveness when using this tool will be avoided. We also acknowledge that the sample was biased towards patients with hematological cancer and that future studies are needed to compare its applicability across different diagnostic groups.

## Conclusion

We developed an instrument to assess patient satisfaction with comprehensive cancer care in Germany. The questionnaire showed satisfactory psychometric properties. Futures studies are needed to validate these preliminary findings.

## Supplementary Information

**Additional file 1.** The German Satisfaction with Comprehensive Cancer Care (SCCC) questionnaire.

## Data Availability

The data and material that support the findings of this study are available on request from the corresponding author.

## Code availability

The code that supports the findings of this study are available on request from the corresponding author.
