# Supplementary material for: Development and preliminary psychometric investigation of the German Satisfaction with Comprehensive Cancer Care (SCCC) Questionnaire
Source: Health Qual Life Outcomes. 2021 May 17;19:147. doi: 10.1186/s12955-021-01784-y (PMC8130117; doi:10.1186/s12955-021-01784-y)
Supplement: Supplementary file 1 — Additional file 1. The German Satisfaction with Comprehensive Cancer Care (SCCC) questionnaire. [file 12955_2021_1784_MOESM1_ESM.docx]

**Supplementary file 1: The German Satisfaction with Comprehensive Cancer Care (SCCC) questionnaire**

Dieser Fragebogen bezieht sich auf Ihre Zufriedenheit mit der onkologischen Behandlung. Bitte beziehen Sie sich dabei auf Ihre persönlichen Erfahrungen, die Sie während Ihrer onkologischen Behandlung gemacht haben. Bitte kreuzen Sie jeweils nur eine Antwortmöglichkeit an.

Die folgenden Fragen beziehen sich auf Ihre Zufriedenheit mit den medizinischen Aspekten der onkologischen Versorgung.

| *Wie bewerten Sie…* | | **sehr schlecht** | **schlecht** | **mittel-mäßig** | **gut** | **sehr**  **gut** |
| --- | --- | --- | --- | --- | --- | --- |
| **1** | …die Sorgfalt und Zuverlässigkeit, mit der Sie von Ihren Ärzten untersucht und diagnostiziert wurden? |  |  |  |  |  |
| **2** | …das Fachkönnen und die Erfahrenheit Ihrer Ärzte? |  |  |  |  |  |
| **3** | …die Sorgfalt und Zuverlässigkeit, mit der sich die Ärzte angesichts Ihrer Krebserkrankung um Sie kümmern? |  |  |  |  |  |
| **4** | …die Erklärung des medizinischen Vorgehens und der Tests, denen Sie unterzogen werden sollten? |  |  |  |  |  |
| **5** | …die Aufmerksamkeit, die Sie von Ihren Ärzten in Hinsicht auf Ihre persönlichen Anliegen/Bemerkungen erhalten haben? |  |  |  |  |  |
| **6** | …die Empfehlungen hinsichtlich einer vorbeugenden und gesunden Lebensführung? |  |  |  |  |  |
| **7** | …die Freundlichkeit und Zuvorkommenheit der Ärzte? |  |  |  |  |  |
| **8** | …die Aufmerksamkeit, welche die Ärzte Ihnen sowie Ihrem körperlichen Befinden zukommen lassen? |  |  |  |  |  |
| **9** | …die Aufmerksamkeit, welche die Ärzte Ihnen sowie Ihrem psychischen/seelischen Befinden zukommen lassen? |  |  |  |  |  |
| **10** | …die Achtung und der Respekt, welche die Ärzte Ihnen gegenüber zeigen sowie der Diskretion in Bezug auf Ihre Privatsphäre? |  |  |  |  |  |
| **11** | …die Fähigkeit der Ärzte, Sie zu bestärken und zu unterstützen? |  |  |  |  |  |

Die nächsten Fragen beziehen sich auf Ihre Zufriedenheit mit den Informationen, die Ihnen von Ihren Ärzten/-innen bereitgestellt werden.

| *Wie bewerten Sie die Menge und Qualität der erhaltenen Informationen …* | | **sehr schlecht** | **schlecht** | **mittel-mäßig** | **gut** | **sehr**  **gut** |
| --- | --- | --- | --- | --- | --- | --- |
| **12** | …zu Ihrer Erkrankung? |  |  |  |  |  |
| **13** | …zu möglichen Folgeerscheinungen Ihrer Erkrankung? |  |  |  |  |  |
| **14** | …zum gesamten Behandlungsprozess? |  |  |  |  |  |
| **15** | …zu den verschiedenen Behandlungsalternativen und deren Folgen (z.B. Chemotherapie, Strahlentherapie, operative Einsätze)? |  |  |  |  |  |
| **16** | …zu möglichen Nebenwirkungen der Behandlung(en)? |  |  |  |  |  |
| **17** | …zum Umgang mit den körperlichen Schmerzen? |  |  |  |  |  |
| **18** | …zu bestehenden psychosozialen Unterstützungsangeboten (z.B. Beratung, psychoonkologische Betreuung, Seelsorge, Sozialdienst, Gruppenangebote, etc.)? |  |  |  |  |  |

Die nächsten Fragen beziehen sich auf Ihre Zufriedenheit mit dem Zugang zu psychologischen und/oder psychosozialen Unterstützungsangeboten, falls Sie diese in Anspruch nehmen möchten.

| *Wie bewerten Sie…* | | **sehr schlecht** | **schlecht** | **mittel-mäßig** | **gut** | **sehr**  **gut** |
| --- | --- | --- | --- | --- | --- | --- |
| **19** | …die Möglichkeit, eine Form der psychosozialen Beratung zu erhalten, um sich bei Bedarf über Unterstützungsangebote zu informieren? |  |  |  |  |  |
| **20** | …die Möglichkeit, psychologische oder psychoonkologische Unterstützung zu erhalten, falls Sie diese benötigen? |  |  |  |  |  |
| **21** | …die Möglichkeit, bei Bedarf eine sozialrechtliche bzw. wirtschaftliche Beratung zu erhalten? |  |  |  |  |  |
| **22** | …die Möglichkeit, eine seelsorgerische Unterstützung bzw. Begleitung zu erhalten, falls Sie diese benötigen? |  |  |  |  |  |
| **23** | …sofern Bedarf besteht, die Unterstützung bei der Therapeutensuche (für Sie oder Ihre Angehörigen)? |  |  |  |  |  |
| **24** | …die Unterstützung beim Erlernen von Selbsthilfe-Strategien im Umgang mit Ihren seelischen und körperlichen Belastungen? |  |  |  |  |  |
| **25** | …die Möglichkeit, an Selbsthilfegruppen oder therapeutengeleiteten Gruppensitzungen zu spezifischen Themen teilzunehmen, wenn Sie diese wünschen? |  |  |  |  |  |

Die nächsten Fragen beziehen sich auf Ihre Zufriedenheit mit der psychologischen Unterstützung durch das Stationspersonal.

| *Wie bewerten Sie…* | | **sehr schlecht** | **schlecht** | **mittel-mäßig** | **gut** | **sehr**  **gut** |
| --- | --- | --- | --- | --- | --- | --- |
| **26** | …die Möglichkeit, während Ihres stationären Aufenthaltes mit jemandem über Ihre gesundheitlichen Probleme und Ihr Wohlbefinden zu sprechen, wenn Sie dies benötigten? |  |  |  |  |  |
| **27** | …die Möglichkeit, während ihres Aufenthalts zuhause mit jemandem über Ihre gesundheitlichen Probleme und Ihr Wohlbefinden zu sprechen, wenn Sie dies benötigten? |  |  |  |  |  |
| **28** | …die psychologische Unterstützung, die Ihnen die Ärzte während Ihrer Behandlung zukommen ließen? |  |  |  |  |  |
| **29** | …die psychologische Unterstützung, die Ihnen die Krankenschwestern bzw. das sonstige Stationspersonal während Ihrer Behandlung zukommen ließen? |  |  |  |  |  |
| **30** | …die Zuwendung, die Sie vom medizinischen Personal insgesamt erfahren haben? |  |  |  |  |  |

Die letzten beiden Fragen beziehen sich auf Ihre allgemeine Zufriedenheit der Versorgung im Rahmen Ihrer onkologischen Behandlung, sowohl mit der medizinischen (die direkte Erkrankung und Behandlung betreffend) als auch der psychosozialen (Besprechung von Ängsten, Niedergeschlagenheit, finanzielle oder rechtliche Fragen etc.) Versorgung.

|  | | **trifft überhaupt nicht zu** | **trifft eher nicht zu** | **trifft teils teils zu** | **trifft eher zu** | **trifft voll und ganz zu** |
| --- | --- | --- | --- | --- | --- | --- |
| **31** | Ich bin mit der von mir erhaltenen medizinischen Versorgung insgesamt sehr zufrieden. |  |  |  |  |  |
| **32** | Ich bin mit der von mir erhaltenen psychosozialen Versorgung sehr zufrieden. |  |  |  |  |  |
